# How do informal information sources influence women’s decision-making for birth? A meta-synthesis of qualitative studies

**DOI:** 10.1186/s12884-017-1648-2

**Published:** 2018-01-10

**Authors:** Ruth A. Sanders, Kenda Crozier

**Affiliations:** 1 Norfolk & Norwich, University Hospitals NHS Foundation Trust, Colney Lane, Norwich, Norfolk NR4 7UY UK; 2 Norwich Norfolk, UK; 3 University of East Anglia, Norwich Research Park, Norwich, Norfolk NR4 7TJ UK

**Keywords:** Meta-synthesis, Birth preparation, Informal information sources, Decision-making, Childbirth, Pregnancy

## Abstract

**Background:**

Women approach birth using various methods of preparation drawing from conventional healthcare providers alongside informal information sources (IIS) outside the professional healthcare context. An investigation of the forms in which these informal information sources are accessed and negotiated by women, and how these disconnected and often conflicting elements influence women’s decision-making process for birth have yet to be evaluated. The level of antenatal preparedness women feel can have significant and long lasting implications on their birth experience and transition into motherhood and beyond. The aim of this study was to provide a deeper understanding of how informal information sources influence women’s preparation for birth.

**Methods:**

Seven electronic databases were searched with predetermined search terms. No limitations were imposed for year of publication. English language studies using qualitative methods exploring women’s experiences of informal information sources and their impact upon women’s birth preparation were included, subject to a quality appraisal framework. Searches were initiated in February 2016 and completed by March 2016. Studies were synthesised using an interpretive meta-ethnographic approach.

**Results:**

Fourteen studies were included for the final synthesis from Great Britain, Australia, Canada and the United States. Four main themes were identified: Menu Birth; Information Heaven/Hell; Spheres of Support; and Trust. It is evident that women do not enter pregnancy as empty vessels devoid of a conceptual framework, but rather have a pre-constructed embodied knowledge base upon which other information is superimposed. Allied to this, it is clear that informal information was sought to mitigate against the widespread experience of discordant information provided by maternity professionals.

**Conclusion:**

Women’s access to the deluge of informal information sources in mainstream media during pregnancy have significant impact on decision making for birth. These informal sources redefine the power dynamic between women and maternal healthcare providers, simultaneously increasing levels of anxiety and challenging women’s pre-existing ideations and aspirations of personal birth processes. A lack of awareness by some professionals of women’s information seeking behaviours generates barriers to women-centred support, leaving an experience expectation mismatch unchecked.

**Trial registration:**

CRD42016041491 17/06/16.

## Background

### Birth is a peak experience

Birth is recognised as an exceptional and life changing experience for women, a journey of transition and re-framing requiring sensitive support and assistance, impacting both short and long-term wellbeing of the entire family unit [1, 2]. Women negotiate a complex array of decision-making processes involved in preparing for birth. This involves aspects of care including different approaches toward birth preparation, choice of birth environment, exploration of mode of birth, all framed within women’s individual perceptions, unique aspirations and wishes.

### Decision-making in healthcare

Preparing for birth is an individualised and fluid process, dependant on women’s unique perceptions and needs, influenced by differences in cultural, religious or spiritual background. The methods employed by women in preparation for birth vary depending on demographic differences including age, relationship status, educational level, socio-economic position and societal environment as well as parity and previous birth experience bearing strong influence [3, 4].

It is widely accepted that models of care and place of birth influence the range of clinical and health implications for women [5], as well as altering the types of preparation women choose to undertake [6]. Decision-making in healthcare is motivated by factors including elements of problem-solving and informed choice, requiring a collaborative dynamic of women-carer relationship [7, 8] until a consensus is reached through deliberation and consideration.

The need to weigh individual choices and make informed decisions about care options can emerge as women attempt to problem-solve issues arising through pregnancy, although decision-making also occurs in the absence of problems [9, 10]. Indeed, the notion of informed-choice transpires through the exploration of possible routes of action, each decided upon through contemplation and reflective process, depending upon individual levels of acceptable risk [11, 12]. Shared decision-making between women and carers has positive benefits for maternal health, forming an expected element of the antenatal relationship [13, 14], although there are reportedly barriers to this exploration of elements of care between women and midwives [15]. The objective of this meta-synthesis is to gain a deeper understanding about how women use the vast array of media available outside the health, professional or ‘organised’ arenas to assist their preparation for birth and how these informal information sources may influence their decision-making as reported by the women themselves. Informal information sources are defined in this study as those sources of information which sit outside the professional midwifery healthcare or midwifery institutional.

### Historical context of antenatal preparation

There is a plethora of knowledge on the ways women prepare for birth, with much of the literature focusing on conventional and professionally-led methods of preparation. This literature [16, 17] assesses the impact of different models of preparatory resources and women’s experiences by gauging levels of satisfaction reported by parents regarding readiness for birth [18–21]. Historically, antenatal education has been championed as a way of professionals imparting knowledge to prospective parents enabling them to prepare for the experiences encountered from conception to early parenthood.

Despite thorough attempts to prepare women using different methods of antenatal education, much of the literature focuses on differences between educational models (standard practice/routine care versus new model being introduced) [17, 22] with general effects, including levels of preparedness parents feel in approaching birth, remaining unclear [17–19, 22]. The reduction of access for multigravid women and constraints for face-to-face contact with midwives result in diminished personal opportunities of contact with known care providers; arguably driving women to seek alternative information sources. There is a dearth of literature concerning the process through which women gather informal information pertaining to birth, and which resources women access outside professional relationships. The midwife-mother relationship is changing at the level of interaction due to an increase of freely available information in digital media form [23]. Women want to make decisions on their own terms, determined by their individual needs and not necessarily rely on largely generalised information which midwives offer during routine schedules of care.

### Technology and e-health changing midwifery practice

Pregnancy and birth related information is readily available from health-care sources, in a context of personal/ professional interaction, as well as through leaflets, websites and healthcare accredited sources. The instantaneous nature of multi-media and technological-focused health culture contextualising midwifery practice further these information seeking behaviours, requiring midwives to work within a sphere of ever-increasingly complex, diverse and continuously evolving technological texts including apps, internet platforms and televisual depictions. The relationship of information giving between women and healthcare providers has changed as a result of rising e-health accessibility [23, 24], although women are seemingly reluctant to share their information findings with their midwives. Indeed, although women’s informational internet searches appear prompted in part by discussion with their midwives [25], some studies suggested that less that 50% of women disclose information they had retrieved from the internet with midwives [23, 25], with many women reporting varying levels of trust and reliability with the information found. This use of digital medias in an advice capacity has led some professionals to question whether to ‘prescribe’ appropriate, accurate sources of information. However, this discounts women’s desires to use digital media to navigate complex pregnancy related decision making and choices for birth.

Portrayals in popular televisual media depict a technological approach to birth, breaking down the historical position of birth as a taboo subject, becoming openly accessible. The frequency of media representations means birth is increasingly discussed with wider external social circles than it has been ever before, shaping women’s expectations from a position further removed from individual circumstance and personal history. This can profoundly negatively impact women’s expectations of the upcoming experience, but can also modify expectations of their ability to navigate labour with representations delivering an often negatively dramatized depiction of birth, a type of ‘one inevitable scenario satisfies all’ portrayal.

Through a dynamic process of information-seeking, various mediums are encountered or specifically accessed including the internet, television documentaries and visual media, newspaper reports, childbirth magazines, mobile phone apps, and social media and peer support involving the discussion of birth stories with women collating what they perceive to be relevant knowledge to assist them in reaching their individual decisions and intentions for birth.

This vast collection of informational elements forms the basis of decision-making of women’s self-constructed ideas, creating expectations of the forthcoming childbearing experience in a notional and unknown future. This becomes a driving force, continually striving to reach a complete level of understanding that quenches their thirst for information, mitigating the unforeseen and unpredictable potentiality for birth [3, 23, 26–29].

## Methods

Meta-synthesis is acknowledged to be an effective way of collating, interpreting and representing synthesised qualitative data, arguably the most recognised format of synthesising qualitative studies [30, 31], with processes of the method documented by many authors [32, 33]. This meta-synthesis is based on meta-ethnography, influenced by Noblit and Hare’s [32] theoretical framework, using an iterative narrative approach.

Because of the interpretive nature of a meta-ethnography, it is understood and acknowledged that a reinterpretation of the various studies may glean alternative readings of this phenomenon of study.

### Developing the meta-synthesis question

Forming the meta-synthesis question happened in stages. Initially this comprised a thorough examination, exploring literature surrounding antenatal preparation. Scoping of literature was undertaken identifying all forms and aspects of antenatal preparation offered, ascertaining the influencing factors which form foundational components women use for their individual decision-making.

This led to the understanding that a gap existed in the literature concerning qualitative research investigating informal information sources and their impact and influence on women’s decision making processes. Although certain papers explored specific elements of women’s information-seeking influential in birth preparation, particularly usage of the internet including apps and social media discussion groups, the literature indicated that these areas have been assessed independently of each other rather than as a comprehensive group encompassing additional elements from women’s social circles such as birth stories and narratives from which women draw conclusions about birth preparation. The meta-synthesis question was therefore defined as:

**“How do informal information sources influence women’s decision-making for birth?”**

To truly capture those complex and fluid elements of women’s experience, the term ‘informal information sources’ was considered by the whole research team broad enough to capture all the individual sources of data (the internet, television documentaries, visual media, newspaper reports, childbirth magazines, mobile phone apps, social media and birth stories) which had not previously been synthesised. This offered an opportunity to form a holistic understanding of the interwoven nature of how women utilise different cultural mediums.

### Search strategy

A systematic search strategy was developed, generating initial terms by investigating literature focusing on women’s experiences of antenatal preparation, the variety of forms offered, and women’s perception of these forms interventions. This developed further once the literature had been scoped by the primary researcher (RS), with a renegotiation of search terms to more accurately pinpoint publications concentrating on women’s utilisation of informal information sources and their influence upon women’s development of decision-making and birth preparedness. The search strategy was readjusted at this point to make sure all relevant data was captured, and to limit the vast number of results which explored specific elements of antenatal decision-making thought too specific for the scope of the study.

Publications were limited to those in English language using qualitative methods. No limitations were imposed on the basis of publication date or country of origin.

Search terms were based on an interpretation of the PICOS framework: problem/population, intervention, comparison, outcome, study type [34, 35].

Searches of the databases Allied and Complementary Medicine Database (AMED), Applied Social Sciences Index and Abstracts (ASSIA), Cumulative Index to Nursing and Allied Health Literature (CINAHL), EMBASE, Medline, PsychINFO and SCOPUS, were conducted between February 9th 2016 - March 15th 2016. Other databases were also searched including Box of Broadcasts, British Film Institute, British Education Index, Cochrane Library (Database of Systematic Reviews), Educational Resource Information Centre but no papers were deemed suitable for inclusion either due to relevance to subject or nature of methodology.

Because of the disparate nature of informal information sources, the search strategy took a two-fold approach. Population, outcome and study type formed the foundation of the search, with four further categories of informal information sources terms providing subcategories searched for individually to ensure clarity, which were then collated to generate a complete and overarching set of papers from each database (see Table 1.) It is acknowledged that database indexing can be problematic when searching for qualitative studies, and so citations were also searched with assistance from supervisors after elimination by abstract.

**Table 1:** Search strategy in MEDLINE

S1-S4 – first and ‘core’ structure of the search
S1 TX pregnant women OR expectant mothers OR women preparing for birth
S2 TX birth OR childbirth OR labour OR labor OR parturition
S3 TX qualitative* OR focus group OR interview OR phenomenolog* OR grounded theory OR narrative analysis OR descriptive analysis OR thematic analysis OR ethnography
S4 TX Information seeking behaviour OR choice OR decision making OR anxiety OR women’s experience OR women’s perception OR birth plan* OR women’s preparedness for birth
S6- S9 added individually to the core search to generate results
S6 TX Internet OR internet forum OR digital media OR social media OR blog OR podcast OR webcast OR mobile applications OR smart phone OR mobile phone technology
S7 TX TV OR television OR reality television OR documentary film OR television documentary OR movie OR film OR cinema OR motion picture OR visual media OR radio OR entertainment
S8 TX Newspaper OR newspaper articles OR magazine OR magazine articles OR self help books OR childbirth literature OR pregnancy literature OR childbirth magazine OR pregnancy magazine OR pregnancy books OR childbirth books OR news reports
S9 TX Birth stories OR birth narratives OR birth Storytelling OR childbirth stories OR childbirth narratives OR childbirth storytelling OR oral tradition OR social networks OR peer support

## Results

### Literature search

This initial process of study selecting was a developmental progression, seeking to capture an exhaustive spectrum of qualitative papers to provide a rich and comprehensive data pool. Highly iterative in nature, the primary researcher (RS) sought and investigated texts based on women’s perceptions of and interactions with informal information sources.

Once final terms were set, of the 2027 papers identified from the final search across the 7 databases, 617 were excluded by duplication with a further 1310 excluded by title and English language. A detailed review of the abstracts undertaken by RS and second checked in collaboration with supervisors (KC) until a final number of 33 titles was arrived at.

A full text read through of the remaining 33 papers (including 4 unpublished PhD theses) for final consideration was undertaken by first reviewer (RS), and then discussed at length with second reviewer (KC). Discussion between first and second reviewer reached consensus by considering papers against search criteria, and relevance to the question, exploring methodology and the quantity of valid information the paper had to bring regarding its new knowledge to the subject area. Through this process final papers were decided upon to take into the next phase of quality assessment.

Upon second read the whole texts were checked against the National Institute of Health and Care Excellence quality appraisal checklist for qualitative studies [36]. This tool was used because of its capacity to inform decision making by service users and providers. All full texts were then assessed by a second reviewer (KC) to ensure the correct approach was undertaken and appropriate exclusion criteria was adhered to. This next step was initiated with the whole research team agreeing that papers which did not meet a high standard of quality assessment may be taken forward for the final meta-synthesis if they provided insight and developed new knowledge.

### Exclusions

Excluded from the synthesis were conference abstracts, and commentaries. Solely questionnaire based qualitative articles were also excluded, as there is some question about the appropriateness of data derived from questionnaires being sufficiently qualitative in design to meet quality assessment criteria [36]. There were a range of papers focusing on vaginal birth after caesarean papers which were also excluded because their focus was deemed to niche for the purposes of this meta-synthesis.

### Included studies

Articles were included which described or interpreted individual reports of how information derived from informal sources aided decision-making preparations for childbirth. Inclusion was focused on articles which used qualitative methods only, with an emphasis on women’s reported experiences from focus groups and interviews.

### Quality appraisal

**Fig. 1:**
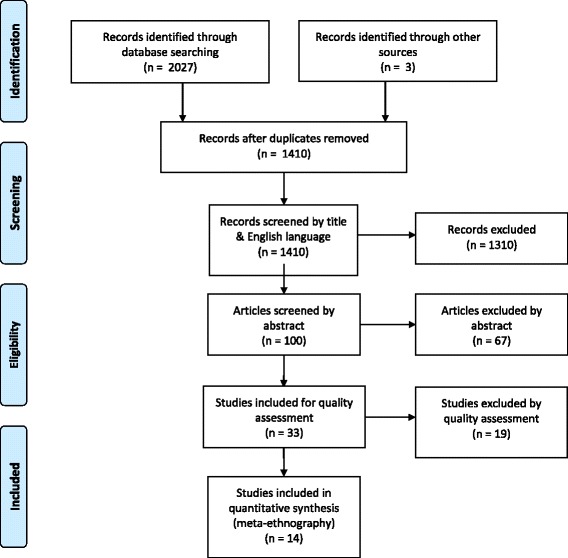
Flow chart of search strategy and outcomes [37]

Final papers (33) were assessed against the NICE Quality appraisal checklist [36]. Because of the nature of qualitative research methodologies, a tool was chosen that could take into consideration the breadth of variety in which qualitative research is undertaken. This tool focuses on the reliability, rigour and richness of data and method, transparency of research design and findings, and allows the reader to contextualise the social processes of the material included. The grading system (Table 2) was determined through rigorous discussion following close independent reading of all papers by first (RS) and then second reviewer (KC). 19 papers were then excluded for various reasons including limited or insufficient data, limited relevance for area of interest and methodological and analytical limitations (Fig. 1) [37]. This resulted in 14 final papers being included for qualitative synthesis (Table 3) [36].

**Table 2:** Quality assessment tool

++	All or most checklist criteria is met, and where have not been met conclusions are unlikely to altar.
+	Some of the checklist criteria have been met, where they have not been met or not adequately described, conclusions are unlikely to altar.
–	Few or no checklist criteria have been met and conclusions are likely or very likely to alter.

**Table 3:** Table of Paper Characteristics (below)

AUTHORS	TITLE	AIMS	METHODS	POPULATION/PARTICIPANT	ANALYSIS	QUALITY ASSESSMENT TOOL	FINDINGS & CONCLUSIONS
Carolan M 2007 Australia	Health literacy and the information needs and dilemmas of first-time mothers over 35 years	To highlight information based dilemmas for women over 35.	1:2:1 interviews 35 weeks, 10–14 days postnatal and 6–8 months postpartum 3 focus groups	22 first time mothers > 35 years, antenatal/ postnatal 19 Women	Thematic content analysis	**+ +** Study design and methodologically appropriate. Useful insight into area of interest. Elements of analysis unreported.	Mothers were given large amounts of clinical information, despite common perceptions of empowerment these women often found the amount of information overwhelming.
Dahlen H, Barclay L, Homer, C 2008 Australia	Preparing for the First Birth: Mother’s Experiences at Home and in Hospital in Australia,	To explore experiences of first-time mothers who had given birth in Australia.	In depth interviews postnatally.	19 women 19–37 interviews lasting 20 mins – 3 h 17 primip, 2 multip Interviews From 6 to 26 weeks (mean 15 wks) Postnatal	Grounded theory One category from the overall analysis ‘preparing for birth’ was the content of this article.	**+ +** Study design and methodology appropriate.	Women cite that the period of preparation for birth has significant influence on and being an important part of their entire birth experience. Women who planned home births felt more prepared and better supported than those planning hospital births.
Fenwick J, Staff L, Gamble J, Creedy D, Bayes S 2010 Australia	Why do women request a caesarean in a normal healthy first pregnancy?	To describe Australian women’s request for cesarean section in the absence of medical indicators in their first pregnancy.	An explorative descriptive approach interview guide based on previous work	14 women 1:2:1 interviews 45–60 min postnatal had caesarean in the last 5 years	Thematic analysis	**+** Methodologically cogent Data not particularly rich.	Fear, safety, control and devaluing the female body and vaginal birth were contributing factors to women’s decision for caesarean birth.
Fenwick J, Hauck Y, Downie J, Butt J 2005 Australia	The childbirth expectations of a self-selected cohort of Western Australian women	To explore/describe women’s expectations of labour and birth and to identify influencing factors	qualitative study using an explorative descriptive design and techniques associated with constant comparison.	202 women pregnant or birthed within the last 12 months. 19–45 yrs. mainly middle class	Thematic analysis	**+** Data not particularly rich Analysis, remained at the descriptive level	Particularly influential on women’s expectations of childbirth were private and public discourses of birth exemplified by books magazines and stories from mothers and sisters.
Flemming S, Vandermause R, Shaw M 2014 USA	First-time mothers preparing for birth in an electronic world: internet and mobile phone technology	Uncover the meaning of how mothers self prepare with electronic media	sequential mix of two qualitative designs: focus groups of professional for preliminary study 1:2:1 interviews for secondary	All low income 7 first time mothers 1:2:1 postnatal interviews 45 mins to 2 h field notes mostly unplanned birth (6/7)	primary hermeneutic (interpretive) design one-to-one in-depth interviews from a purposive sample (*n* = 7) of young first-time mothers (FTMs) hermeneutic (interpretive) phenomenological approach	**+ +** Rich description of data Methodologically appropriate and coherent.	FTMs were preparing for birth ‘what ifs’ with electronic media based on what is ‘known’ about birthing. Mothers became educated but also this increased levels of fear and anxiety.
Freeze R 2008 USA	Born free: Unassisted childbirth in North America.	To explore reasons women, choose to have unassisted birth. Exploring why women make this choice; the knowledge sources they privilege; how they understand the concepts of safety, risk, and responsibility, and their complex and sometimes contradictory relationship with midwifery.	interviews and personal correspondence, surveys, and archives of internet discussion groups and forums. Internet was the primary means of gathering participants. Telephone interviews 30–90 min 4 professionals interviewed followed the discussions on many UC and birth-related Yahoo groups read over 100,000 posts	sixty-one survey responses 17 telephone interviews mostly middle class	Thematic Analysis? – not clearly stated but approach was documented as thematic	**+ +** Methodologically appropriate, very rich use of data	The process of freebirth is complex and an understanding of why women free birth is needed to identify why some women are driven away from certain models of care offered by professionals. The study highlights the abuses and limitations of current paradigms of care UC bridges the gap drawing from professional practices but acknowledging women’s need for autonomy in the birthing process.
Lagan B, Sinclair M & Kernohan G 2011 UK	What Is the Impact of the Internet on Decision-Making in Pregnancy? A Global Study	To build on studies to explore women’s experiences and perceptions of using the internet for pregnancy related information and influences this has on decision making.	Interpretative qualitative Thirteen asynchronous online focus groups across five countries Pilot study tested first	92 women who had accessed the Internet for pregnancy-related information over a 3-month period.	Framework analysis	**+ +** Methodologically appropriate Insightful regarding stories	The internet has a marked impact on women’s decision making across the entirety of their pregnancy, highlighting a great need for information.
Miller A 2009 USA	‘Midwife to myself’: Birth narratives among women choosing unassisted homebirth	Detailed women’s narratives created by women choosing to birth unassisted	127 unassisted homebirth stories sourced from Yahoo and google 10 face to face in depth interviews to check findings coherence	10 participants	Grounded theory Constant comparison	**+ +** Strong insights into internet and us of books Methodologically appropriate but some lack of detail in analysis	Women rely on both medical and midwifery models and wider understandings from unassisted childbirth circles to formulate agency around birth. They reference formal models of care whilst seeking to liberate themselves from it.
Munro S, Kornelson J & Hutton E 2009 Canada	Decision-making in Patient-Initiated Elective Cesarean Delivery: The Influence of Birth Stories	Exploring birth stories and cultural knowledge that women use to inform decisions for elective cesarean without medical indication.	Explorative in depth interviews with 17 women One branch of the total research findings are represented. 7 sites 2003–2005	17 primiparous women interviewed by 2 researchers 30–90 min	Grounded theory Constant comparison	**+** Methodologically appropriate Some insights but limited in terms of data richness and analysis	Women drew heavily from social and cultural knowledge in forming their decisions to birth by caesarean.
Regan M, McElroy KG, Moore K 2013 USA	Choice? Factors That Influence Women’s Decision Making for Childbirth	Filling the gap in knowledge investigating factors that influence women’s decisions about birth	Mixed method 13 focus groups over 12 months	49 primiparous women 21–36 yrs. majority white	Consensual Qualitative Research method	++ Methodologically appropriate Insightful and rich data about sources of information	Four major categories were found but only birth stories and attending a birth have lasing effect on influencing birth choices
Rodger D Skuse A, Wilmore M, S. Humphreys S Dalton J Flabouris M & Clifton V.L 2013 Australia	Pregnant women’s use of information and communications technologies to access pregnancy-related health information in South Australia.	Examines how pregnant women living in South Australia use information and communication technologies (ICTs), principally Internet and mobile phones, to access pregnancy-related information.	35 semistructured interviews conducted as part of the larger ‘Health-e Baby’ project, a qualitative study	35 women aged between 19 and 40 yrs.	unstated	+ methodologically limited –no discussion of analysis data richness limited some useful insights	Shows that ICTs have great potential for health promotion communication high levels of access not easy to predict personal choices pregnant women make for mode of communications they access, prefer & trust
Seibold C 2003 Australia	Young single women’s experiences of pregnancy, adjustment, decision-making and ongoing identity construction.	To examine young pregnant women’s experiences of embodiment, identity construction decision making and how these are influenced.	Explorative descriptive study using feminist principles	5 women 17–23 yrs. interviews both antenatal and postnatal telephone interview at six months post birth. Women also kept diaries	Techniques of grounded theory were used	**+ +** methodologically appropriate rich data	All women welcomed the physical changes of pregnancy. Acceptance of pregnancy was assisted by supportive families, friends and sympathetic healthcare professionals, as well as exposure to opinions via classes, information and educational opportunity.
Song F, West J, Lundy L, Dahmen N 2012 USA	WOMEN, PREGNANCY, AND HEALTH INFORMATION ONLINE: The Making of Informed Patients and Ideal Mothers	To explore how white middle class women use the internet during experiences of conception, pregnancy and childbirth to ascertain how internet usage challenges, and medical paradigms shape women to make decisions	Part of a descriptive study on the information-seeking habits of women in five areas of early mother- hood: conception and fertility; pregnancy; labor and delivery; child’s feeding and nutrition; child’s health and safety products.	32 mothers interviewed November 2008 and March 2009 24 to 36 years all but one Caucasian 1/3 multiparous women complex and un-complex health experiences	Grounded Theory Feminist approach	+ Level of analysis unreported in places, methodologically appropriate	Internet enables socially privileged women to perform an informed patient role ad demonstrate their competencies as mothers.
Weston C, Anderson J 2014 UK	Internet use in Pregnancy	Perceived Value of internet in pregnancy from the view points of midwives, pregnant women and postnatal women.		Thirteen midwives, seven antenatal women and six postnatal women three focus groups and seven in-depth interviews.	Appropriate internet use was valued by all groups	+ Useful despite methodological weaknesses useful discussion of ‘apps’ although analysis appears on a surface level	Appropriate internet usage during pregnancy was positively valued by all groups. Greater collaboration between midwives and pregnant women is required to enable access to consistent, verified internet information which can be used appropriately and confidently.

### Design of Analysis and Selected Studies

Text engagement took an evolving approach, identifying underlying concepts and themes emerging from individual papers. Papers were re-read (RS) and explored, seeking to identify how emerging themes were related to the area of interest. This commenced thematic analysis, aiding identification of themes compared across the data set. This was checked by a second reviewer (KC) to ensure an appropriate level of reflexivity was carried out and themes were translatable across the data set. A thematic style was discussed between reviewers initially seeking out connections between texts.

A data extraction tool was collaboratively developed and applied to each paper to extricate major concepts and themes. This involved direct quotes from first order concepts (women’s quotes), selected on the basis that they provided a comprehensive understanding of the variety of women’s feelings and thoughts from each paper in relation to relevance of the research question.

Second order concepts were then entered into the data extraction forms using sections of original papers which epitomised authors core findings and nuances of their interpretations. This was done deliberately to explore similarities and differences between first and second order concepts. This progression was checked (KC) to ensure trustworthiness and credibility of the data extraction.

**Fig. 2:**
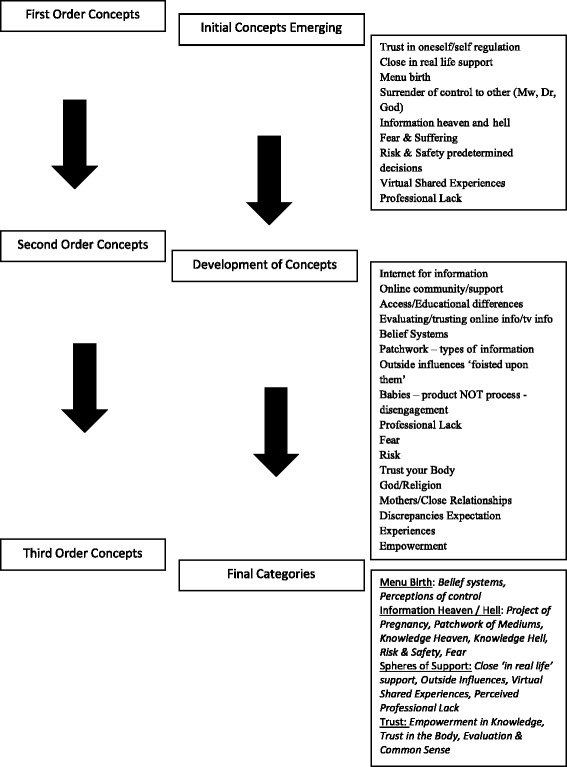
Meta-Synthesis Development Process

Data extraction forms were then deconstructed into individual first or second order quotes. These were differentiated by the use of different text (first order themes were italicised and second order were not) making it possible to link individual data pieces to original papers by use of colour coding. To ensure a sound meta-ethnographic approach there was no fixed starting point or paper from which all others were compared, this was a deliberate decision to make sure that all data captured was decontextualized. First order quotes were randomised and categorised allowing for a more reflexive position emerging themes on a quote by quote basis (see Fig. 2). This same process was replicated for author interpretations (second order quotes) generating further concepts with patterns amongst the data being reviewed and renegotiated using open coding.

Groups were combined, formulating a narrative iteration enabling translations and initial synthesis between texts, enhancing reflexivity within the process. Core overarching themes were established with analysis moving fluidly between data and theory, seeking to separate first and second order concepts in order to ensure no systems of hierarchy remained within the data set, perceiving each subset with equal gravity. This led to synthesising studies in an organic, fluid process akin to axial coding translating core concepts across papers, drawing together final thematic categories and third order concepts.

### Findings

The fourteen papers included in the final synthesis represent findings from four countries, two from the United Kingdom [28, 38], five American [27, 39–42], six Australian [26, 43–47] and one Canadian [48] published over a nine-year period between 2003 to 2014. One paper was an unpublished PhD thesis [39]. Samples ranged from 5 to 202 women with one study including one mans views. Predominantly primagravid women with a range of methods employed to explore aim of the papers. The findings are presented in terms of the four emergent themes: Menu Birth; Information Heaven & Hell; Spheres of Support; and Trust. The key themes are divided into subthemes which explore key concepts.

### Menu birth

#### Belief systems

Women express a sense of expectation and desire toward pregnancy and birth which appears to arise from an internal understanding pertaining to an unarticulated ‘knowing’ of what will happen. This stance if considered as a “menu approach” to their forthcoming process of pregnancy and birth, is engaged with as if it were possible to pre-plan their desired experience, organising the type of birth with a resolute kind of determination.

*‘Basically, I just wanted that opportunity to like choose the way my labour was going to progress but if I did need to make choices like about pain relief that I would be supported by the midwives and my doctor’* [44].

Although this amalgam of embodied self-knowledge and from whence it derives is not explored within the papers, women’s understanding of personal belief accrued from various elements; narratives, images and informational encounters; forms each individual perspective, existing outside and prior to the experience of pregnancy [43, 44]. This foundational understanding of the process of childbearing is the bedrock upon which all future information-seeking and decision-making is constructed.

Women do not arrive to pregnancy as empty vessels, devoid of information. Rather their experience in approaching the process is formulated from this foundation, regardless of whether it originates from a projected ideal dream of ‘birth as peak experience’, or a sense of trepidation based on cautionary narratives. ‘*…want it to be as natural as possible, no drugs and I don’t want an epidural.’* (Megan) [47].

Trusting their chosen ideation forms a confirmation bias whether its caesarean or physiological birth which is decided upon, informing and driving women to pursue their desires once pregnant. When the notional idea of pregnancy becomes a reality, women’s internalised and perceived ‘knowing’ of an unrealised process becomes the backdrop upon which is projected desires demands and aversions.

‘*Early on, I made a list of all the factors and elements I did or did not want to be part of this experience. My main focus was on creating an absolutely uninterrupted, undisturbed process of birthing, controlled entirely by me. I wanted no input from anyone else while giving birth. I wanted no suggestions, no instructions, no checking, measuring, or labeling. I had total confidence that I would have a safe and normal birth.’* [40].

The transition from internal belief to external expression becomes the articulated menu of the process of childbearing.

#### Perceptions of control

Control is explored across the papers from a variety of standpoints moving outwards from the women in terms of relational proximity; personal, religious, familial and institutional. A powerful sense of control is gained by women’s information seeking behaviours [28], whether this is surrendering their own bodies to the process of birth, or surrendering the illusion of control over to something/someone external and outside themselves.

Women’s thoughts encompass a trend toward exerting or surrendering levels of control over the process of labour and birth. Notions of control manifest in an array of forms, often being equated with an idealised birth experience and a way of ensuring that the desired menu of factors is provided.

Control motivates different elements of women’s decision-making in relation to the desired menu of birth experiences to be achieved; including environment, care provider [26, 43]; mode of delivery [39, 40, 45, 48] and pain management [27, 47]. This connection between the overarching belief system and how the menu manifests through control is clear from women’s discussion.

‘*a controlled environment with all the bells and whistles and the experts there to do whatever needs to be done’* (Vera) [45]

*‘drug free and not a caesarean as little intervention as possible’* [44]

*‘I didn't read anything for 9 months except birthing books, I swear. I mean, I just studied it I knew it so well that the birthing classes suggested that I become a teacher. By the time I was in preparation for her birth, I had a lot of very, very positive expectations around the birth. I envisioned love around me and my own environment. To have all the things that I wanted, like music and the candles and the aromatherapy and my sister and husband and Amy [midwife].’* [43]

Many participants express their desire to control birth from a complex and dualistic standpoint. Women discuss approaching labour with a ‘knowledge’ that the process is seemingly controllable but one which easily transmutes into an uncontrollable event. That birth is inherently personal and unique is understood, yet women view labour as an event which is both internally and externally controllable in terms of their perception of their bodies. This is expressed through ideations of the self, both as controller of their bodies and the labouring body as an object to be controlled, navigating the experience as well as being a passenger to the process undertaken.

Although predominantly focused on first time mothers’ experiences, some discussion derives from the desire to avoid repeating a previous birth experience, influencing choice of mode and healthcare provider or in cases, unassisted homebirth [39, 40, 45].

This culminates in the necessity that someone take control, expressed in a form of self-surrender, exerting self-control via the choices women make for birth involving environment, pain management, pre-decided mode, or through the surrendering of elements existing externally to the construction of the self. Participants from Fenwick et al., [45] reported the belief that caesarean birth offered a ‘higher level of control’, whilst Freeze and Miller’s [39, 40] participants, focused on free birth philosophy, felt this enabled them to not only control what they themselves did and felt but when and how things occurred, prepared their environments themselves, therefore sculpting the interactions which happen within these settings. Freeze suggests a connection between women choosing to free birth and those choosing elective caesarean; that regardless of mode the outcome is driven by the desire to avoid repeating traumatic experiences through increasing levels of control:

*‘I personally find the attitude of, ‘my (insert care provider here) will take care of everything no matter what so I don’t have to prepare,’ to be INCREDIBLY irresponsible.’* Adelaide [39].

There are nuances between decisions made in the process of pregnancy and those relating to choices during birth. Women reported they had ‘*little control in labour when things went wrong*’ [47]. The concept of being ‘ready for anything’ because unpredictability is the only absolute, was echoed across papers [26, 44]. This acceptance of unpredictability and the desire to be ‘in charge’ [44] empowered women to experience control, avoiding feelings of disappointment or the sense that they had let themselves down if their menu birth was not achieved.

This created illusion of a ‘controllable process’ develops with the involvement of others, negotiating surrendering control over to women’s facilitators.

‘*I trusted them. I handed control of myself over to them. I was completely in their hands’. (*Deanne) [45].

Closest in relation to self-surrender appears to be the presence of God, and women readily relinquish power to a greater force directly related to women’s existing belief systems: *‘This baby was unplanned but there was no question that I would not have it, I had an abortion at 18 and that really upset me. This baby was God’s gift.’* (Megan) [47].

Surrender control becomes connected to women’s imagined predetermined birth, and there is an element of self-empowered certainty which occurs in this transference of control to God:

*‘I just figured it was God’s will whatever happened.’* [40]

*‘They call it “do-it-yourself” home birth, right? Who’s really “doing it,” anyway? Does a doctor bring that baby out, or the Lord? But you know that already! It just seems such a revelation when you finally discover it for yourself.’* [40]

Included in the discussion of a greater force, are husbands. In several papers, husbands appear to hold positions of authority over the process as head of women’s families, in the stead of health care professionals. This is discussed within the wider religious context of unassisted birth and predominantly limited to those specific papers [39, 40]. *‘God had answered me. He wanted to show me a different way of doing things. A way that involved faith and not fear, and from that night on we decided that when we had children they would all be born His way. My husband said, “After all, God didn’t seem to be worried that His own Son was born in a stable with only Mary and Joseph present.” I couldn’t argue with that!’* Faye [39].

*‘I had [my husband] check the position and the station of the baby’s head and to make sure I was complete, which I was, and to also do a quick Doppler scan of the baby’s heart beat.’* [40]. Institutional control is discussed across papers, with health care professionals situated as keepers of control and knowledge, able to harness the process of birth:

*‘You’re not actually driving the car. You want the obstetrician to drive the car. That’s what you are paying him for’. (*Megan) [45]

*‘People were saying, ‘Oh my god, you can paralyze and this and that.’ I said, ‘Don’t worry about it. They know what they’re doing, they’re not paralyzing, you can get hit by a truck tomorrow, too.’ There’s so much misinformation about c-sections.’* [48]

*‘When it’s conflicting...if people are putting different things...it does get a bit confusing but, erm, then I’ll ask my midwife.’* [41]

Lagan et al. [28] suggest that a powerful sense of control is gained by women’s information seeking behaviours and this is found across most papers. Many women appear to approach the project of pregnancy and childbirth with the same method of organisation as a ‘work project’ by gathering information and then formulating a plan [26]. Inevitably there are overlaps between a sense of control and the loss of control experienced through the acquisition of information. This will be further explored in the subtheme *Knowledge Hell*. Information seeking then contributes to the way women seek to control those elements outside of themselves.

To summarise, the concept of the menu birth is encapsulated by how women retain control through the choice of whom to surrender their experiences to, the level of control they are able to maintain and choosing the facilitators who best fits within the belief system of birth menus women are seeking.

### Information Heaven & Hell

*‘You just don’t have any idea what you need to know, and no one can really tell you that, you just have to find it out as you go along…’ (*Agatha) [26].

#### The project of pregnancy

The fundamental driving force behind women’s searching and sifting of information is to find sources which either support or challenge their belief systems in order to make informed and appropriate decisions for themselves.

Information sources accessed include the internet, books, magazines, visual media, social media forums, with high levels of internet usage reported in the wider literature at an ever increasing rate [23, 25, 49, 50].

At this turning point, when dreaming about the process of pregnancy becomes a lived reality, women report the amount of information amassed leads to feelings of empowerment, experiencing an enhanced level of control, as well as having the potential to increase anxiety without slaking the thirst for information, which exacerbates the need for further exploration. This is compounded by the ever-present awareness of time constraints within pregnancy. Thus, women’s approach of ‘project management’ allows for a more logical examination of information in contrast to the dreaming before emotionally committing themselves to their individual position.

Women appear to desire information which confirms and therefore justifies the rational for their philosophical approach when engaging with the project of birth, acknowledging the ramifications arising from both found or supplied information; and the breadth of cultural mediums within which this information resides. *‘I find if I’m worried about something and I can read about it in a book or a magazine and I like what they have to say then I feel much more settled. Happier.*’ (Megan) [47]

*‘my main priority (was that) the baby came out healthy no problems’.*

*‘expectations were really just to have a healthy baby at the end’.* [44]

A selection of women discussed birth and the journey of labour as as a process by which they can get a baby – the product of the process [45]. This was found predominantly in papers focusing on caesarean birth with the emphasis on babies’ health as paramount, and women’s experience having secondary importance. The focus for these women is about babies’ health as the end product goal– rather than the actual process of birth. Fenwick et al. [45] describe the act of ‘getting the baby’ as the dominant factor, that the experience is not considered but merely a means to an end; rather than the experience of ‘having a baby’ as the challenge to explore. Women reported that they felt that they had *‘Nothing to contribute to get the baby out’* [45: pg 398]. Women’s involvement in the process of birth was removed or minimised, becoming divorced from their role in giving birth ‘*I don’t care if it comes out my nose’* (Katrina) [45].

Women attempt to put their wishes aside, martyring their desires for birth by placing the baby’s importance above their own.

*‘It didn't bother me. It’s just as long as he was healthy.’ (Nancy, hospital birth)* [43]

Freeze [39] suggests that when women freely disclose their wishes for a fulfilling and rewarding birth experience this can be met with accusations from outside, opening women to being ‘accused of being selfish, narcissistic, or callous about the well-being of their babies’. For these women birth is entirely defined in terms of the baby, with women unable to articulate any sense of personal connection; caesarean birth considered as a procedure in which the baby was removed, a type of *‘means to an end’* [45]. Carolan [26] reports that the disconnection and focus on the end product has lasting repercussions, with women finding themselves ill-equipped for parenting and neonatal care. Women’s transition into motherhood and renegotiation of their identity construction as parents is also related to their unwavering focus on birth, leaving little time to consider the effects of becoming parents.

*‘My initial concern was telling my parents. I was no longer with my boyfriend and did not want him to be part of it [the pregnancy]. Once I knew Mum and Dad were OK with it I was relieved and settled. It was only later it started to dawn on me: This is going to change my life forever. I’m going to be a mother. How will I manage.’* (Karly) [47]

*‘It was like suddenly she was here and I knew nothing. Before she was born I worried a lot about other things... ‘is she going to die’ I worried about the cord choking her... the business with the placenta, and how it can be left behind... everything I read seemed to point to some other danger, so although I read a lot, I kept myself very busy so that I wouldn’t worry too much... I didn’t let myself think... then when she was born I couldn’t sort of, couldn’t give my 100% attention to the baby, and I didn’t have any idea how to look after her.’* (Jennifer) [26].

The result of placing the baby at the centre of birth becomes a discrepancy and experience/expectation mismatch. Women discuss their feelings with a lack of emotional connection – expressing ‘anticipation’ and ‘relief’ [45], and although women appeared to be positive about their choices they voiced experiencing an unforeseen ‘sense of ‘nothingness’ [45]. ‘*You get up feeling no different go to the hospital a couple of hours later I’ve got a baby in my arms you feel like you’re at a shop getting milk or something’* (Katrina) [45]

Women in Carolan’s paper [26] disclosed postnatally that they subsequently viewed their sole focus on birth and the relationship this had to information as detrimental, and only through acclimatising into motherhood and relating to their child could appreciate their inability to see past the birth experience. This sentiment was also found in other papers across the study.

*‘It’s a little bit different from what you expect all your life. Once she was born and we saw her, the doctor said ‘there you go we’re finished’ and I thought is that all? Is that it? I had a lot of trouble with attachment. I can remember looking at her for several days afterwards and thinking ‘where did you come from?’* (Deanne) [45]

#### Patchwork of media

Women report that midwifery information remains influential throughout their decision-making for birth, with antenatal classes [26, 41, 43, 47] and personal encounters cited as important [28]. However, although women do access direct midwifery information there are barriers to this professional client/patient dynamic and even though possibly preferable to some women [38], all women also seek knowledge from additional sources. Women across the studies prepared with variable levels of intensity sourcing diverse materials through an equally diverse assortment of media.

Information which historically would have been provided solely by women’s close communities and family members [42] appears to have evolved, divided into categories in terms of the relational proximity the information has to the seeker, what Fenwick et al. [44] term as the public/private discourses surrounding birth preparation, streaming in through a wide spectrum of discrete or public platitudes, direct and indirect forms.

Aside from professional advice (midwife, obstetrician or General Practitioners [G.P]), public sources which appear supposedly factual include leaflets, books, the internet and visual media. These contrast with private and highly personal information including stories from family members and friends, social media, internet forums, and attendance at a birth.

Certain sources bridge both groups, blurring the private/public discourse by providing information as well as depicting the process of birth as entertainment, allowing for certain elements of gamification. These include television programs and mobile telephone apps which whilst reportedly a valued resource [46, 51] apps particularly have the added complexity of ludifying pregnancy [52].

Books are frequently cited as valuable sources, often accessed upon recommendations from support networks [40–42, 48] including specific pregnancy guidebooks, as well as certain midwifery titles and niche publications relating to free birth, with magazines and journals cited at considerably lower frequency.

Reading materials are not confined to books; written accounts and information are also sourced via the internet. All papers in the study noted the internet as highly influential, anonymity playing a fundamental and positive feature [28]. Volume, the ease of accessibility and convenience all play a crucial role with immediacy and abundance of information sources resulting in positive and negative responses to the content discovered.

*‘The Internet is fast and immediate. I didn’t have to wait until office hours or until someone returned my phone call. I didn’t have to go to a library or bookstore. I could look up information while at work or at home, anytime.’* (Laura, USA) [28].

This is particularly relevant in relation to the internet searching where general search terms are at the whim of internet search engines:

*‘When I was a kid, my parents were big ‘investors’ in encyclopaedias ... Google is the encyclopaedia of this century. The Internet contains so much information that can be accessed in seconds.*’ (Infinity, Australia) [28]

Women amass copious amounts of information across the data which was met with various responses:

*‘. . .if you’ve got something on your phone that tells you what’s going on. . .you are more likely going to look at that than rummage through pamphlets trying to find something when you can type in physically what you want to know and it tells you.’* [46]

*‘lot of reading….first pregnancy it was all sort of judged on what I’d read in books’* [44]

*‘There are a number of books I’ve been doing my reading from during my pregnancy. Each of them are good in different ways. The more books I read the better I feel. Some books cover some things that others don’t. Some also conflict which I think shows every pregnancy is different*.’ (Lorraine, diary entry) [47]

Compared with other sources, the internet is viewed by many participants as ‘the font of all knowledge’ [28], women reporting that it offers the wider range of views. Books and magazines are considered narrow in focus, quickly out of date, and costly offering no current accurate information.

The internet is used mainly in either seeking specific pieces of information, or for accessing women’s birth stories on social media, once again the private domain of preparatory work expanding out into an impersonal domain.

*‘I was on the Internet all day. Like, any sign I was wondering if that was a sign that I could be pregnant, thinking every month I was pregnant. Or even how to get pregnant, like how long the sperm was in you. I was just constantly on the Internet looking at how to get pregnant and what I should be doing.’* [42]

*‘I would get concerned with certain health issues, and look up things, I just wanted to know everything about it. I looked up a lot on folic acid because I was worried that I was having too little. I bought a lot of books and read them, but sometimes I looked for more information, like I would read something and ‘Oh that’s interesting, but didn’t give me enough’ and I’d tend to look up more on the Internet*.’(Jane) [26]

Drawing on other women’s birth stories regardless of source seemed to serve the powerful function of attempting to engage with the sense of unknowing, women reporting that is was *‘Impossible to be fully prepared’* [26] and that by collecting a multitude of stories were able to dream into the uncertainty of birth, trying out differing scenarios to mitigate the discomfort of the unknown [27, 39, 43].

*‘child-birth is an unknown thing you never know what is going to happen’*

*‘Parenting magazines. I just read them…you read all the birthing stories’* [44]

Discussions of visual media are reported as having longer lasting impact on women than other media [27, 44, 48] whether vaginal or caesarean birth is viewed. The information which appears to be a closer representation to ‘real life’ the more visceral the experience for the perceiver, having a more profound, longer lasting influence.

‘*we looked up on YouTube C-section videos ‘cause neither of us knew anything about it.’* [27]

*‘[o]nce I got in the operating room ... I saw the images of a woman in my head being cut open, and I was just, oh, my gosh, that’s what they’re doing to me. I was just scared in general. I should have read about it instead, I guess. It was the blood.*’ (Lucille) [27]

#### Knowledge heaven

The immediacy of sought information and free access regardless of socio-economic standing changes the onus of becoming informed back to women, shifting the educational power paradigm from professionals creating the possibility for self-empowerment. Decisions are made in relation to inclusion or avoidance of this tapestry of cultural narratives.

*‘That was very important for me, to be as knowledgeable as I could be about the process that my body was going through. I did read a lot... books, on the Internet, information from the hospital.*’ (Harriet) [26]

This creates a safe and unfettered space where women can explore their options, taking control and shaping decisions about how informed they want to be, with women making decisions about birth drawing from both embodied personal knowledges as well as more medically accepted traditions to inform their decisions [48].

‘*You know, years ago when people didn’t have such free access to information you just did as you were told and that was, you know, that was the way things were gonna happen and that was it, whereas I think now it does allow people to make more informed choices or at least ask the questions around other choices and other ways to do things.’* [41]

This means that those birth practices which were considered fringe and unconventional, such as unassisted childbirth, become familiar and more mainstream and a viable choice through increasing levels of media exposure [39], offering broader choices to a wider population.

*‘When I found out I was pregnant, I knew that this time I wanted a homebirth. I had just moved to Mississippi, so I didn’t know where to find a midwife. . . . There just wasn’t anybody. Then one day I was online looking at pregnancy stuff and found Laura Shanley’s website on unassisted birth. I couldn’t believe it! I mean, these stories, they were so—they were just like nothing I’d ever seen. I knew then that was what I was going to do.’* (interviewee) [40]

#### Knowledge hell

The enormity of available resources can however, lead women to feel overwhelmed by the barrage of information. This overloading can prompt women to abandon their search; the sheer volume of opinions, thoughts, tropes and memes, either halting or completely derailing the ability to filter the information into any helpful semblance of cognisance. The trajectory of their decision-making process undergoes a moment of reflection and evaluation, to take stock and consolidate their position in relation to the current informational surfeit.

*‘You’re just inundated with lots and lots of information, I’m sick of reading, everything you read mentions other things you never even thought of....’ (*Annie) [26]

Some women commented on the difficulty when sifting through layers of information to uncover relevant facts which can enable effective and informed due to varying amounts of misinformation.

*‘a lot of pregnancy books are geared towards one ideal or another, and it’s hard to get information that’s just factual.’ (Participant 25).* [41]

Women also reported concerns regarding a lack of background knowledge or framework with which to position this newfound information, often causing high levels of anxiety and confusion, prompting a type of literal fight or flight response needing to encounter more resources or retreat from information all together:

*‘...now I wish I had adopted an ostrich type principle, where you don’t worry so much about it, where you don’t want to be so informed*.’ (Jane) [26]

Therefore, while informal information allows women to be empowered, engaging proactively in decision-making [28] it also propagates fluctuating fears and a certain level of distrust.

#### Risk & safety

*‘I was a bit overwhelmed with all the things that could go wrong’.* (Karly) [47]

Mothers spoke of their specific views of the information they collect and how this information gathering led to ‘perceptions of risk and vulnerability’ [26, 45], exploring perceptions of risk and safety and how risk becomes rationalised and incorporated along the decision-making process. The papers which discuss caesarean birth in the absence of medical indication [45, 48], suggest women generally reported either filtering out or ‘switching off’ to risks, that somehow because the surgery was controlled and planned for, women didn’t consider known risks to be applicable to themselves. This is rationalised by women as an issue of choice, and in their consumerism of this mode of birth they reach a sense of informed decision-making and choice, at the same time as often lessening the prospect and potential severity of surgical birth.

‘*Risks? So what? There is risk in everything you do and to me, having a caesarean section presented me with less risk than the vaginal. I felt I was bypassing the risk and so did my doctor’.* (Jane) [45]

Papers discussing unassisted childbirth [39, 40] considered women’s choice to birth unaided as a means of disengaging from the current paradigm of risk averse maternity culture to determine their own spectrum of acceptable risk and safety. Rather than discussing risk in terms of the uncontrollable elements of birth that might go wrong, they spoke of losing their sense of agency and authority over their own experience, as well as the influence of staff actions, drugs and technologies which accompany institutional forms of care on offer.

*‘in preventing the stress, interventions and routines that come with having a ‘professional’ at the birth who looks for things to go wrong, I know that I will be providing the best atmosphere for things to go right.’ Ernestine* [39]

Women acknowledged that there was a constant state of searching out anything which could potentially propose risk but that this needed moderating:

*‘If you want to keep looking...if you do too many searches...Paracetamol...I ended up going further down the searches so...there was the NHS, you know, Paracetamol’s fine, Paracetamol’s fine. It was almost like you can get in danger of this macabre- like thirst for finding out there must be something wrong...and of course coming across a danger study that had linked Paracetamol use with, erm, schizophrenia in later life, you know, I was like “yes I found it” [laughter].’* [38]

Safety can be found in choice, with some women reporting an emphasis on avoiding certain eventualities of birth such as Fenwick et al.’s [45] paper citing women’s desire to not ‘*get all ripped up’* (Deanne) transferring issues of safety to their unborn babies: ‘*Your child’s head doesn’t need to be squished like that, and it would not have happened had she had a vaginal bypass’* (Amelia)’.

Women discussed feelings of safety in their choice of environments:

*‘They [hospital staff] were there if I needed help, so I felt pretty secure there [hospital].’ (Nancy, hospital birth)* [43]

*‘You'd have a better chance of it being natural, a natural type of birth at home, than you would in hospital.’ (Leanne, home birth)* [43]

‘*I watched a movie on Netflix about hospitals and like giving birth in hospitals ... they told me like once you have an epidural, you can’t move.’* [27]

Type and presence of care providers is discussed, noting the strong influence of birth stories which asserted negative outcomes, as well as the risks of adhering to advice from unregulated sources such as the internet.

*‘I didn’t go through the midwives only because it was my first baby and I was wary. So I thought I would go through a doctor.’ (Bess, hospital birth)* [43]

‘*when my brother was born he had a broken nose and a broken collar bone and I was in fetal distress when I was born’*. (Sophie) [45]

*‘The chat rooms…it put me off…one woman I saw on there had been bleeding for hours and she was going on there instead of phoning the doctor or midwife and I just, that’s, that’s quite dangerous…’* [38]

Although recognized by women, risk was not often overtly spoken about in terms of decision-making, rather some women reported a reversed type of arrival at decisions, where information which didn’t fit into their rational of choice was disregarded.

*‘Quite frankly I still don’t know how many risks there are with caesarean section. I wouldn’t have wanted to know about the risks I had made my decision’*. (Dee) [45]

#### Fear

*‘birth is scary and frightening’* [44]

The pervasive nature of images, accounts and information emphasising the ‘horror story’ narrative running through cultural discourses surrounding birth, and the damaging nature of decontextualized snap shots which then become dramatic representations within certain mediums, have lasting effects on women. The result of the previously discussed overload of information is that women report feeling frightened and anxious [27, 28, 44].

This is further compounded by the varied capacity women demonstrate for making sense of the information found. This gives rise to increasing levels of anxiety and fear of feeling unprepared, apprehensive about search results, as well as raising issues they had not yet considered [26, 27, 47]. General discussions of fear which related to ‘giving birth’ found women describing the process as ‘*terrifying, petrifying, frightening, scary, worried’* [44].

*‘I was absolutely petrified of the whole ordeal of the birth itself through the vaginal canal and also a lot of it had to do with the loss I felt, the loss of dignity to the mother at the time of birth*.’ (Annette) [45]

Some anxiety discussed, is based on women’s prior experience with fear arising out of a wish to avoid repeating what went before, or a distrust of hospitals and institutional care models [39].

Fear is inextricably linked to ideas of pain through several papers, seeming to manifest in the exploration of labour discomfort. As the unknown becomes known through the accessing of information, that which is in a process of being uncovered remains anticipated, with attention on the unmanageable and uncontrollable elements of birth. This results in women feeling under threat from the forthcoming experience, responding to the process of laboring and birth from an external standpoint, existing outside their sphere of embodied knowledge, giving women no context other than the information which they are processing from various sources.

*‘Because I saw the pain! . . . I was like, I’m about to go through this, and I can’t do it. If I hadn’t seen her go into birth, I probably would be like, I can do it. It’s nothing. But I saw it, and I can’t.’* (Participant 26) [41]

According to Flemming et al. [27] nearly all participants experienced visual media as invoking ‘extreme fear’ or ‘pure terror’ responding to an array of birthing videos or internet shows.

*‘[i]t didn’t really help prepare me. It ... made me, – anxious to get it over with because I wasn’t really prepared for the actual birthing part. I thought it was going to be terrible. With TV shows, birth is filmed like they do when they show a rape. The lights dim down, the music changes and show becomes more dramatic. Your heart starts racing.’* (Dana) [27]

Even those women who chose not to access visual information made the choice out of a motivation of self-preservation, knowing that the information would be in some way destructive, one woman describing her avoidance of televisual representations so as to ‘*Pretty much not to be horrified before I go the hospital, you know?’* (Tiani) [27]. This is reflected in other papers across the study, regardless of delivery mode, with a dramatised version of childbirth heightening the cultural sense of pathology, which confirms and compounds women’s preconceived understanding that labour is a fearful event.

Information heaven and hell can therefore be considered in two phases. Although there is a widely reported feeling of information heaven arising from women’s freedom to explore all available options via the plethora of accessible sources which women can choose from to fit into their existing framework; the overriding feeling about the resulting content of information found, increases fear, anxiety and the need to only search further for reassurances about the information they have collated.

### Spheres of support

Women’s desire and search for supportive individuals and a wider sense of community and commonality is a chord running through all of the papers. Connection and support is sought on different emotional and relational strata, manifesting in both the ‘real life’ and virtual mileau.

#### Close ‘in real life’ support

*‘My mum had five children my mum told us about being born she had four home births and one hospital birth, so she told me what it was like and what she has gone through so I think that was a big influence.’* [44]

Women cite mothers as featuring highly on the influential spectrum. For younger expectant mothers, acceptance of pregnancy and the positive emergence of their sense of identity as ‘mothers to be’ related to support from family members, particularly their own mothers [47]. There is some suggestion that for older pregnant women whose peers predominantly had older children, the ‘void’ which presented itself from a lack of discussion and reassurances from their own mothers was replaced with a strong reliance on reading large amounts of literature [26]. Accessing mothers was a way of validating women’s own ideas and experiences, explored in an intimate and private pre-existing relationship, with some papers reporting those stories from mothers and sisters having the highest impact on women [43–45].

*‘to think, six months ago I was barely discussing anything with my mother. I knew she was a wise woman, but I didn’t want her involved in my business. Now I want her very much involved*.’ (Karly) [47]

The recounting of maternal narratives from mother’s pregnancies and birth experiences are intended as encouragement and comfort for many women, aiding some women’s capacity to visualise birth in relation to expectations and perceptions regarding mode of delivery [45].

These narratives, historically situated and emotionally weighted by familial propinquity, can be fraught with complexities and opinion rather than factual unbiased information, and were regularly found to be challenging and in some cases detrimental [43, 48]. These birth stories can inadvertently and unintentionally become part of what Fenwick et al. [45] describe as ‘vicarious trauma’ promulgating negative and fearful inferences resulting in an iatrogenous and corrosive effect on women’s emotional facility to engage with the forthcoming event.

‘*When I was born I almost killed my mother. It was a twenty-four hour labour and she had two hundred and seventy internal stitches’* (Sharon) [45]

Women disclosed the importance of guidance from other close personal relationships when navigating questions of information resources and birthing venues.

*‘I mean, there’s a ton, there’s so much information out there when you’re pregnant and there’s so many different books to read. I’ve had several friends who have recommended many of these books to me that have had natural childbirth or been with midwives, so maybe that’s why I’m getting these particular kinds of books.’* (Participant 32) [41]

*‘My friend. it wasn’t that she wanted to scare me, but I was asking her to explain to me exactly how she delivered and she’s my best friend and she told me exactly.’* [48]

The power of these maternal and sororal stories and birth narratives cannot be understated with many studies calling for further exploration of the implications of these private dialogues.

#### Outside influences

Being culturally visible means that pregnant women are vulnerable to becoming public conduits for others understanding of the embodiment of pregnancy and motherhood, a focal point for others upon which to project or affirm their own schema, individual value judgements and understanding of what the future holds for expectant women. Women reported unsolicited advice being ‘foisted’ upon them from outside [26, 41]. Liminal in nature, pregnancy is transient provoking time sensitive motivations in others to impart opinions, meaning the social constructs which ordinarily apply to everyday interactions in a socialising context become suspended. One woman had to ‘cease relationships with people that were important [to] her because [she] just can’t hear what they have to say anymore’ [41]. The embodiment of parturiency leaves women exposed and in a highly malleable state, compelling them to seek a shared connection for reassurance and support.

#### Virtual shared experiences

‘*I wanted to make sure what I was experiencing, was not unique to me, that other women had experienced the same thing.’ (Noelle, Canada)* [28]

The interactions of online forums and social networks allows women to be publically enfranchised whilst retaining a sense of personal anonymity. The sense of freedom this allows is widely reported [27, 28, 40, 42, 46, 48]. While many women do utilise ratified websites such as healthcare and governmental information sources, this is outweighed by the use of commercial and social media [46]. The urge to connect with other women in order to glean insight into birth, especially for those seeking alternative or niche birth choices such as unassisted childbirth, was voiced with expectant mothers finding safety and acceptance on the worldwide web which may not be present in everyday spheres of support [28].

Community spirit is recreated in virtual space, with the range of connection reported as largely beneficial for women, with immediately accessible support and guidance from online ‘friends’.

*‘My ‘real-life’ friends didn’t have any experience of pregnancy, so the Internet forums I visited provided me with ‘friends’ who had been through it before, or were going through the same experiences as myself.*’ (Nicky, UK) [28]

*‘The Internet. mostly the Internet and the people. Talking to different people. it was experiences because I wanted to hear it firsthand from others. And each has their own opinion or own vision about it so. then you make your own conclusions and you make your own choices on all of this.’* [46]

This level of connectivity gives women entrance into a space of consideration unencumbered by potential disapproval or critique, allowing for a different sense of privacy in which to explore options [28]. The alternative side to this freedom is that women can encounter extremes of the birth spectrum, with stories offering alarming as well as reassuring narratives.

*‘You’re the one I’m going to believe, help me out here ... they give you like worst case scenarios and best case scenarios; how common is this really?’* [27]

Online distance also enabled other aspects of pregnancy choice to be expressed, and although predominantly supportive, women reported that this wasn’t always the case, and just as in face to face communities there were instances of interactions becoming passive aggressive in nature.

*‘It’s so competitive as well...this baby is not even here yet and already people are kind of comparing “oh what’s yours doing?” “Oh my bump, I play classical music to my bump” [laughter]...on things like Mumsnet, it’s quite smug I think about how wonderful their children are and I think... it’s just a bit patronising really.’* [40]

However, these annoyances were outweighed by the positive responses women felt they received from their peers and the facilitation that these encounters rendered. The range of national contexts and private versus publically funded practice did not seem to have a marked difference in responses from women.

#### Perceived professional lack

*‘Health professionals often don’t have the time or inclination to explain things in the detail you would like.’* (Leah, Australia) [28]

A general perception of professional lack is suggested throughout women’s information seeking journeys. This lack of professional guidance and support is experienced in a wide array of forms. Although reportedly this is partly due to the time constrains imposed by current heath care providers and their systems, this constitutes part of a more complex malaise. The lack of an individualised and meaningful relationship with a known care provider leaves women with an unstable foundation and this subsequent sequalae brings about a lack of trust in not only the health care professional but in the information they provide [28, 38].

*‘. . . it does surprise me, I actually have to say, that the process of the OB/G visits haven’t been as personal as I thought that they would be. I thought my birth plan was something that you sit down with your OB/G and discuss but I’m figuring out this is something that I [have to do] on my own.’* (Participant 47) [41]

Routine schedules of care imply a predetermined and professionally orchestrated agenda rather than a woman-centred approach for professional contacts. Women sought to fill this information void by self-generated research.

*‘Appointments these days seem very few and far between (every 6 weeks, right up to 36 weeks), and I often have concerns and queries in the meantime which can be found out about, and often resolved, using the Internet. Even with such a fab mid wife, though, appointments are often very short, and although for her pregnancy is a completely everyday matter, for us expectant mothers it is a huge and important part of our lives for 40 weeks*.’ (Kerry, UK) [28]

Allied to this, there is a reported discrepancy between information received from midwives and that of physicians, suggesting that more options were discussed by midwives than their clinical counterparts [41]. This variation was not only based on type of professional but also on the demographic of participants. It could be argued that this is a positive approach to individualizing care, and that information should be women-centred and respond to each woman’s needs. It is noteworthy that women at either extreme of the childbearing age spectrum are considered to have different informational needs by professionals, appearing to receive an increased level of information or to be curated for differently [26, 48]. The experiences of women across the study report a distrust in professional’s ability to facilitate and enable their birth choices.

‘*None of them could say whether I could have a water birth. They weren't very supportive of the idea*.’ Tracey [43]

As well as feeling unsupported, women also commented on how midwives were disconnected from women’s own concerns. Women suggested that the internet was valuable for generating conversations with midwives, but that midwives should also be accessing internet sources to better comprehend women’s specific concerns.

*‘It would be good to know if the midwives are perhaps, erm, looking up on their own at the most top common concerns that their patients have and seeing what is available online so that they’ve got an idea of the kind of stuff that we’re viewing...even if it’s rubbish.’* [38]

Women report hesitation in trusting professional veracity, questioning the advice they receive from doctors and midwives. Historically there is a perception of reliance on healthcare providers, and the emergence of alternative avenues of support and information has eroded the primacy of this position.

*‘If I had not had the Internet and just had to rely on the information the doctor gave you, I really don’t feel confident that that is all there is to the story. I never feel confident that they are going to tell you every single thing that you need to know. So I thought it was my job—if I was going to do this—to learn about it.’* (Joanna) [42]

This apparent lack of connection with professionals, further fuels women’s need for information and connection outside the professional sphere of support.

The relational proximity women felt regarding the type of support experienced had direct impact on the uptake of information incorporated into their decision-making processes.

### Trust

Trust presents in a variety of representations; trust or distrust in professionals, trust in the information sourced outside professional relationships, trust in women’s innate capacity to birth, and the trust in their own decision-making capabilities.

Lack of trust in professionally offered information galvanises women to source intelligence from elsewhere to bridge the knowledge gap which endures.

*‘... My health professional had only one opinion and appointments are short. I found the Internet had different perspectives to offer and more current information.’* (Jeannie, USA) [28]

Additionally, acquiring information from informal sources emboldens women’s sense of confidence, not only in questioning the type of information they receive from professionals, but also their faith in professionals’ willingness to share all information with them [28, 42].

*‘I felt really empowered having such an amazing resource available from my own home ... it put me in control to a degree, and I feel really lucky that I was pregnant in this decade and not in the “old days” where all info came from a medical professional, who often gave only their OPINION and not balanced info ... I found that if I researched a topic, and THEN approached my doctor, I got a more “honest” answer (more detail). Having the ability to research at home helped me to make informed choices during my pregnancy...’* (Rhianna, Australia) [28]

Whilst women expressed feelings of distrust toward professionally provided information, they acknowledged that they were still partially dependent on professional ‘expertise’. Women stated they would access midwives’ advice on issues which they perceived as being ‘serious’ or clinically based [26, 38, 42].

#### Empowerment in knowledge

Women value the independence that autonomous information seeking provides, with ease of access granting women the opportunity to add to their innate individualised perception of parturition [23]. This leads to heightened empowerment, altering the way women are able to communicate their care choices with providers.

*‘Oh, I think people just rolled their eyes and said, “There she goes again,” because I always want to do things myself and be independent. I can’t stand the idea of giving someone else the power to decide what happens to me!’* [40]

Increasing confidence discussing their care arose from being better informed about issues of import to women, rather than professionals being the keepers of knowledge [42]; making it possible for women to challenge the authority of their providers [38].

*‘I know what to expect a bit more from my, erm, appointments because of the internet...so if they don’t do something you can, er...it must be a nightmare for midwives...I think [name of midwife] forgot to measure me like on my first one or something I was like “aren’t you supposed to measure me? Measure my bump?”...I think if you had a shoddy midwife and you were well informed... it would sort of empower you to say, to challenge a bit more to say I don’t think you’re right.’* [38]

Not only are women empowered through informal information to alter the historical professional dominance, some women also appear to place equitable value on informal sources as those gleaned from professional sources.

*‘We’ve come to a certain point where we know a lot more than we would have 20 years ago, and we want to know, the risks and all that, so I find that a really difficult question because I don’t know if I could approach it any other way if my doctor didn’t give me enough information I would’ve got it somewhere else.’* (Jane) [26]

Although they continue to place value in their care provider, women consider that their value is equal to their own capability to resource themselves. This is also connected to previous themes of embodied knowledge, that if information found does not align with women’s perception of birth, then they feel stronger to discard it and continue to seek that which is more confluent to their beliefs [43].

#### Trust in the body

*‘It truly was a life altering experience for me, and the most incredible thing ever. My faith in myself as a mother, as a woman, as a human being, went up 200% after that. I just really and truly believe that my body was made to have babies and my faith in it is so strong now. Its almost impossible to put to words.’*Lee [39]

As informal information is absorbed, women begin to situate themselves in relation to their decisions for birth. There is an increased sense of autonomy which comes out of knowledge empowerment, with appreciation of their own capability to choose how they approach labour.

Women who are present to witness birth in adulthood talk of it as a seminal experience, forming an intrinsic part of their decision-making process. Regan et al. [41] report that nearly 20% of their sample had the opportunity to witness a birth.

*‘I had a powerful experience when I was early on [in my pregnancy]. It was home birth of my friend’s . . . I think for me, deciding to go with home birth had somewhat to do with being there at a home birth. . . .That seems worthwhile: I want to be a part of that. I want that. And so early on, we made that decision.’* (Participant 40) [41]

Women appear to trust their stance resulting from informal information, whatever their expectation of upcoming birth. For some women this resides within their bodies, trusting that birth is a normal physiological process which their bodies capable of. Women questioning their bodies as capable or not to deliver their child, must be considered in terms of their belief and negotiation of whether birth is a normal process or not.

*‘My body, and nobody else, knew the best way to deliver my baby!’* [40]

*my sister and my mum and my aunty and everybody else you know had babies ‘in lifts and all sorts of things so I expected it to be easy’* [43]

This realization was apparent from those women choosing unassisted childbirth. By gaining control via informal sources, women’s beliefs are confirmed, and as Freeze suggests ‘women are becoming anti attendant and their thought processes are that if they trust the process 100% then nothing bad will happen’ [39].

*‘I started out with the plan to have a nice homebirth with a midwife. But after reading all the books, I just thought, hell, I can do this. I’ll just be a midwife to myself!’ (Interviewee)* [39]

*‘I was simply listening to what my body was telling me and following that without question.’* [40]

Other women placed trust in the fact that they would be able to birth but acknowledged that it would be an experience to endure.

*‘I was expecting a very painful and long childbirth and I was expecting quite a lot of medical intervention’* [44]

*‘Awful! I really expected it to be extremely painful because Mum said her labor was 23 hours for her first, which was me, and I was really scared. But it wasn't as bad as I thought it was going to be.’* [43]

Some women absent themselves from the process because of a lack of trust in their ability. By avoiding undergoing the process of labour women’s choice to elect caesarean birth negates the possibility of perceived failure.

*‘She had a second child and had it planned right, so like I called her up and said, ‘When’s the baby due?,’ and she was like, ‘Oh you know, like July 1st at 3:15.’ And I’m like ‘What?,’ and she’s, ‘Oh we’re planning it this time. If I couldn’t do it the first time I’m not doing it the second time.’ And starting from then, I sort of went, ‘Oh, what a civilized way of doing it.’* [48]

#### Evaluation & Common Sense

Women’s ability to asses and filter information about the birth process has a significant impact on what is included in their decision-making for birth. Through the sifting and sorting process they become aware of the either a value or limitation to what they have sourced, as well as knowing when additional information is needed to make decisions. Across the papers there was a lack of comprehensive discussion of quality assessment of the information other than their own perceptions of ‘common sense’ [27, 38, 41, 48] and although some papers showed women’s understanding of the entertainment purpose [27, 28] rather than fact, this was not a deterrent to watching/looking at these sources.

Women determined what was useful when seeking information, acknowledging that the information found was in some ways potentially questionable. Women reported that it was important to discern found information by applying the notion of using ‘common sense’ to conclude which information was of value [27, 38, 48]. However, the ways in which this value judgement was arrived at is not fully explored. The notion of ‘common sense’ implied that women knew how to assess what was valuable or untrustworthy.

‘*Yeah so you kind of have to do a certain amount of self-regulation I think.’* [38]

‘*They put the scariest ones or the worst ones on television. And it didn’t really help prepare me. It just kinda made me, you know, more – I don’t know – anxious to get it over with because I wasn’t really prepared for the actual birthing part. I thought it was going to be terrible.’* [27]

This trawling for common sense is based in process and purpose, however what is often discovered are feelings of communality rather than a commonality of understanding. This communality serves an emotional deficit but does not constitute a rigorous base from which to create decisions. There is a persistent lack of women applying standards of quality to information they interact with. Thus, it is only women’s own ability to critique the incoming barrage of material in relation to their own position that is used to prioritise information for inclusion or exclusion.

Fenwick et al. [44] question whether women are passive recipients to the information or whether they are actively engaged with the consideration of information on offer. There was an understanding from women that the level of quality of information was low, weak, and poorly referenced and that women did not place great ‘trust’ in the information sourced online [27, 46]. Certain papers suggest that the credibility of the teller of information was weighted differently depending on the intimacy of relationship to the women, suggesting that a closer relationship would provide a higher level of trust and credibility to the information given [43, 48]. Women recognise that there are circumstances in which they would choose to search out information and when they know not to, instances connoting that the seeking will unearth undesired information which may not be beneficial.

*‘It’s just that some things...you know when to Google and not to Google’.* [38]

*‘[be]cause you go further anyway cos it’ll come up like...could have this or this and you’re like, click, oh no, need to Google that.’* [38]

Arguably the process of determining credibility increases women’s vigilance to their own experience of pregnancy. The papers demonstrate that beneficial knowledge can be gained from an increased confidence in negotiating informal information, especially that sourced online [28, 38, 42]. This is also based on how women choose to situate the found ‘evidence’ within their embodied knowledge base [48]. Women still struggled with a lack of comprehension and advanced information seeking resulting in concerns not previously considered [26]. The filter of common sense relates to the impact of found information, yet not always applied to knowledge which was considered fear-inducing.

Women trusted that they were able to begin decision-making when they felt that they had arrived at ‘enough’ information. Although not discussed in all papers, Lagan et al. [28] suggest that data saturation was attained when women found the same desired information in different media formats or repeated on alternative sources [28] or when they felt satisfied that they had found the answers they were seeking.

*‘I would stop searching when I thought that I had enough information to make an informed decision/opinion.’* (Susan, Australia) [28]

*‘Stopping searching for me was often more to do with being satisfied that I had learned all I could on the topic (a bit like knowing when you’re full at the dinner table).’* (Tania, New Zealand) [28]

Song et al. [42], also suggests that the process of women becoming informed allows them to ‘prove’ themselves as informed patients, representing culturally favourable ideals of positive mothers as a result, all contributing to women’s decision-making capacity.

To summarise, women use trust as an agent to determine their own capacity to arrive at decisions, a lens to confirm their thoughts and situate themselves in relation to the process of labour and birth. Trust driven courses of information seeking deliver decisions aligning each woman’s beliefs and aspirations, echoing their initial starting point of the menu process toward birth.

## Discussion

The aim of this meta-synthesis was to enhance understanding of the influencing factors women encounter outside professional healthcare relationships during pregnancy leading towards birth. These results indicate that although informal information sources are strongly influential upon women’s choices for their birth, these decisions are not made in isolation. Rather, they become woven into the pre-existing phenomenological understanding women already possess, with women’s decisions developing out of embodied knowledge interacting with large amounts of pervading informal information.

Women do not arrive in pregnancy as empty vessels but instead with a whole host of aspirations and expectations. Women utilise informal information to validate their own choices for birth. Communicating desires with others allows women to generate a supportive environment through exploring the question of ‘is it just me?’. Likeminded communities ensure feelings of security in the knowledge that women are not alone in their choices. This seems to be particularly desired if their birthing wishes sit outside the ‘norms’ of recommended care pathways. Lagan et al. [28] suggest professionals can aid women’s seeking, offering guidance in order to avoid them becoming overwhelmed. However, there is a sense of women’s need to ‘do birth’ themselves with as little intervention, assistance or pain-relief as possible [43].

The synthesis suggests that those matters existing outside women’s predetermined understanding of pregnancy or that threaten to disrupt this understanding are dismissed or disengaged with in favour of ideas which hold to own ideologies. Because of such wide-ranging seeking behaviours, givers of information from all sources are considered. Their influence on women’s final decisions for birth depends on their relation to women’s existing belief systems and where the information lies in terms of distance from their original perceptions. This constitutes a form of reversed informed consent, women having decided on choices for birth long before being confronted with any evidence, also dependant on who is entrusted with input into their birth experience. Childbearing exists within a media informed culture [27], the immediacy of access changing the way women process information. The democratic distribution and dissemination of knowledge impacts women’s autonomous thought processes in association to their decision-making. The speed of gathering information arguably affects how fast women arrive at decisions due to how quickly they are now able to compile and support their own knowledge-base. Through searching for information women become aware of acceptable portrayals of motherhood and emergent pregnancy identities. The concepts of ‘menu birth’ and ‘project pregnancy’ situate the process of childbirth in terms of consumerism, although this varies across papers from different healthcare cultures and countries depending on whether healthcare is publically provided or privately paid for. Although largely dependent on the wider healthcare context within which women experience their pregnancy, ‘menu birth’ is an illusion, with availability remaining dependant on professionals’ facility and propensity to offer all choices equitably. The rush of the product, not only resides in the ‘getting’ of the baby, but becomes translated into the ideally imagined future birth, constructed from the patchwork of mediums women have created. Individualised care is what is sought because women do not experience it in the current system. Women do not necessarily seek evidence but rather to create their own evidence-base. The menu is constructed only from what professionals deem suitable and propitious, not driven by women’s birth wishes. It could be surmised that women are situating themselves to rely less on professional information and to broaden their knowledge of information sources because they don’t trust what professionals have to give. This is a way of making woman-centered decisions in the absence of woman-centered care offered.

Although the study did not aim to explore how women felt about their relationships with professionals’, the choices to seek information were commonly made in response to what they felt was lacking from professional interactions. The predominant feeling of ‘lack’ revolved around how women placed themselves in relation to trusting professional involvement and levels of trust placed in professional expertise as well as the amount of self-control women desired to exert on their experiences. This trust spectrum varies for each woman ranging from wanting professionals heavily involved and ‘in charge’ of care, an idealised notion of ‘shared care’ with professional advice discussed within women’s internal sphere of pregnancy knowledge, or little or no professional involvement in cases such as unassisted childbirth. This was influenced by choice of mode primarily, dictating the model of care – that women paying to have elective caesareans were happy to entirely trust in professionals whereas women choosing unassisted childbirth chose to birth at home with an absence of provider.

This was also dependant on the amount of trust women possessed in themselves and their role in the process of birth, influenced heavily by the media informed paradigm of present midwifery culture. Therefore, women who felt those elements of high importance to themselves were not met by professional interaction then this needed to be sourced externally. Professionals reported many women ‘wanted to know everything’ and this was considered challenging, and met with varying levels of exasperation from professionals, implying that women should be satisfied with what professionals deemed relevant for their care. There was a sense that ‘they [women] have no idea’ and that their ideas are constructed in relation to ‘others’ understanding and experiences of *their* birth experiences. Women’s understanding of their own expectations is to some extent, experienced through perceiving themselves through the views of other people, leading to a phenomenological dissonance and incongruence. It is noteworthy that professional communication with women contains a certain amount of birth dramatization [42], influencing decision-making for birth through the coercive nature of professionals’ recounting horror stories.

This can resound with some women’s preconceived ideas, prompting antenatal catastrophizing behaviours fuelled by the relentless exposure to dramatic birth depictions throughout accessed media. Women’s psychogenic landscape then becomes heavily influenced by a desire to avoid pain, assuage fear and negotiate the persistence of visceral and graphic birth images, removing the unknown and replacing it with a dramatic re-envisioning both from external sources and internalised imaginings from assimilated stories.

The findings in this paper need to be considered in relation to limitations. The evidence synthesised was collected over a relatively short period of time and due to the expanding speed of information accessible to women, it provides only a contemporary view of women’s influencing factors with quickly evolving variable factors. By focusing on experience of women only the experiences of professionals were not fully investigated but is an area which warrants further exploration. The papers synthesised were of variable quality and the methodology applied to the data is open to alternative interpretations. However, the study has many strengths including the methodological possibilities of meta-ethnography offering alternative interpretations in an expanding field of knowledge, and the study design of attempting to synthesise the data by mirroring women’s phenomenological experiences of information gathering including all non-professional resources offers a unique perspective to data-synthesis.

## Conclusion

The interconnected elements of informal information and their collective influence on women results in no *primary* driving force of decision-making. Informal information sources then, influence women’s decision-making as a collective mass, each strand being chosen in an unfolding response to women formulating their informational needs as information is encountered.

Rather, the emphasis remains on women’s ‘having to choose’, weighing up how important each element is based on which sources extol women’s bias and belief systems, supporting their intentions and aspirations for birth.

This meta-synthesis highlights the significant impact of informal information sources on women’s decision-making for birth. Midwives should acknowledge that women are accessing large amounts of information independently and attempt to learn what the women in their care are seeking from the onset of the professional journey rather than merely following the pathway laid out by a professionally determined menu of options. This study offers an opportunity for remodelling professional interactions to bridge the gap which exists in women’s knowledge base, but must be women driven. In order to re-organise services, we must first understand those issues driving women to access informal information. The study shows that women have preconceived ideations of motherhood and it becomes therefore necessary to determine at what point women’s beliefs and perceptions for birth begin to solidify prior to the point of conception. Through facilitating trust the cultural and individual background of each woman must be comprehended, exploring her ideations of motherhood.

It is counter productive, in the facilitation of women’s peak experiences of birth, to have considerable time constraints and lack of flexibility approaching decision-making conversations throughout the pregnancy journey. Facilitating a presence of mindful engagement with women’s current realities is needed from midwives whilst negotiating the need for the exploration of all potentialities. A study of professional linguistics would assist in the formulation of a deeper understanding of how to enable a coproduction of open dialogue between health care professionals and women, yet this can only truly be fostered within a model of trustful continuity of carer.

## Recommendations

The need for an in depth qualitative investigation of mainstream media depictions of birth and sororal storytelling remains, with no reports as yet of the long lasting implications on women of childbearing age but also of those girls and women undergoing their individual burgeoning ideations of birth. Various forms of media will continue to provide various narrative of birth through the lens of drama. It is unlikely that writers and producers of these media will eschew a dramatic lens when creating popular cultural texts around birth. Thus, this will be an ongoing issue for the foreseeable future, in the climate of expanding technologies and access to birth related media and information. It will continue to be important that healthcare professionals provide accurate sensitive and individualised information to resource women toward making their own informed decisions. This would give midwives an improved understanding of women’s expectations redefining women as the keystone of their own experience in the midwife/mother dynamic.

Unless these issues are acknowledged and addressed in current practice, and without standardised policy and further investigation, women will continue to fill their knowledge gap, not necessarily communicating this to professionals. This will allow the disjointed mismatch between expectation and experience to continue undetected and unaddressed by midwives, missing a pivotal opportunity to improve experiences for women and their families.

## Abbreviations

IIS: Informal Information Sources
NICE: National Institute for Health and Care Excellence
